# Membranous nephropathy and lupus-like syndrome after hematopoietic cell transplantation: a case report

**DOI:** 10.1186/1752-1947-4-303

**Published:** 2010-09-10

**Authors:** Kostas Stylianou, Stavros Stratakis, Vasiliki Mavroeidi, Ioannis Petrakis, Dimitris Xydakis, Eleftheria Vardaki, Spyros Stratigis, Kostas Perakis, Theodora Katsarou, Peggy Kanellou, Irene Xylouri, Constantina Petraki, Michael Alexandrakis, Eugene Daphnis

**Affiliations:** 1 Nephrology Department, Heraklion University Hospital, PO Box 1352, 71110 Heraklion, Crete, Greece; 2 Hematology Department, Heraklion University Hospital, Crete, Greece; 3 Department of Pathology, Evaggelismos Hospital, Athens, Greece

## Abstract

**Introduction:**

The kidney is increasingly recognised as a target organ of chronic graft-versus-host disease after hematopoietic cell transplantation in the context of the development of the nephrotic syndrome. Chronic graft-versus-host disease is associated with autoimmune phenomena similar, but not identical, to those observed in various rheumatologic disorders, implicating autoimmunity as an important component of the disease.

**Case presentation:**

We report the case of a 57-year-old Caucasian man who developed the nephrotic syndrome due to membranous nephropathy in association with recurrent chronic graft-versus-host disease, along with a lupus-like syndrome manifested with pancytopenia, hair loss, positive anti-DNA antibodies and sub-epithelial and mesangial immune deposits. To the best of our knowledge, this is the first case reported in the literature. The nephrotic syndrome subsided soon after he was treated with a short course of cyclosporin with steroids. Unfortunately he died seven months later due to a relapse of leukemia.

**Conclusions:**

Our case report confirms the notion that chronic graft-versus-host disease is characterized by the appearance of autoimmune phenomena similar, but not identical, to those seen in autoimmune diseases. The decision for more immunosuppression has to be weighed against the need for preservation of the graft versus leukemia phenomenon.

## Introduction

Acute graft-versus-host disease (aGVHD) has traditionally been defined as a syndrome occurring during the first 100 days following allogeneic hematopoietic cell transplant (HCT). aGVHD occurs in 9 to 50 percent of patients who receive HCT, despite intensive prophylaxis with immunosuppressive agents [1].

Chronic graft-versus-host disease (cGVHD), by definition, appears over 100 days after HCT and is associated with autoimmune phenomena [2]. Auto-antibodies found in patients with cGVHD are similar, but not identical, to those observed in various rheumatologic disorders, implicating autoimmunity as an important component of cGVHD [3].

The kidney is increasingly recognised as a target organ of cGVHD in the context of the development of the nephrotic syndrome (NS) [4-9]. We report the case of a man who developed NS due to membranous nephropathy (MN), three years after HCT, along with clinical and laboratory findings resembling systemic lupus erythematosus (SLE).

## Case presentation

A 57-year-old Caucasian man was referred to our renal ward when he was found to have developed NS. Five years earlier, he had been diagnosed with acute myelogenous leukemia (AML-M2) for which he received induction therapy with cytarabine and idarubicin followed by mitoxantrone and VP-16, with complete response. One year later he underwent HCT from his HLA-identical sister at his first relapse. Their compatibility was complete for HLA A_1,24_, B_8,35_, C_W4,7_, B_W6_, DR_11_, DR_W52_, DR_B1_, DR_B2 _and the transplant came from peripheral blood stem cells. Busulfan and cyclophosphamide were given as a conditioning regimen and cyclosporin plus methotrexate as a prophylaxis for GVHD. Methotrexate was discontinued at day 30 and cyclosporin was gradually tapered until it was stopped at six months. Despite prophylaxis, he developed extensive cGVHD with widespread skin eruption and elevated liver enzymes eight months after HCT. He was then restarted on cyclosporin for two months and prednisone which was discontinued 12 months later when all signs and symptoms of cGVHD had subsided. After cessation of the immunosuppressants, he gradually developed hypertension, proteinuria (2 g/d), mild creatinine elevation (133 μmol/L) and elevated liver enzymes (SGOT 200 U/L, SGPT 207 U/L, ALP 197 U/L, LDH 479 U/L). Reinstitution of prednisone resulted in a clinical improvement but six months after the cessation of steroids he developed NS with anasarca, proteinuria (4 g/d), hypoalbuminemia (1.7 g/dl), and elevated serum creatinine (150.3 μmol/L). An attempt by the treating physicians to obtain kidney tissue by a biopsy was not successful at this time so he was empirically commenced on furosemide (80 mg/d), enalapril (10 mg/d) and methylprednisolone (48 mg/d) with subsequent improvement of proteinuria and his renal function.

One year later, when steroids had been withdrawn due to a worsening cataract, he presented with pancytopenia, alopecia and a relapse of NS. At this point he was referred to the renal ward of our hospital for the first time. On clinical examination, he was apyrexial with pitting edema in both of his legs, loss of hair in his armpits and on his head, and poikiloderma lesions on his arms and trunk. A laboratory investigation showed the following: hemoglobin 9 g/dl; white blood cell count 4800/μl (28 percent neutrophils, 60 percent lymphocytes, 12 percent monocytes, no blasts); platelets 98000/μl; serum creatinine 212 μmol/L; increased liver enzymes (SGOT 97 U/L, SGPT 51 U/L, ALP 235 U/L, LDH 578 U/L); and nephrotic range proteinuria 4.3 g/d. Urine analysis showed increased erythrocytes (20/HPF, 70 percent dysmorphic), few mixed casts (consisting of white blood cells, tubular epithelial cells and rare erythrocytes), few oval fat bodies and plenty of hyaline casts. Virology tests for human immunodeficiency virus (HIV), cytomegalovirus (CMV), hepatitis B virus (HBV) and hepatitis C virus (HCV) were negative. His serum immunoglobulins, C3, C4, rheumatoid factor, anti-mitochondrial antibodies and anti-nuclear cytoplasmic antibodies (ANCA) were all normal. A direct Coomb's test was negative, anti-nuclear antibodies (ANA) were positive (1/320 diffuse staining) and anti-dsDNA antibodies were also positive by enzyme-linked immunosorbent assay (ELISA) (74 IU/ml). However, the crithidia luciliae immunofluorescence test (CLIFT) was negative.

**Figure 1:**
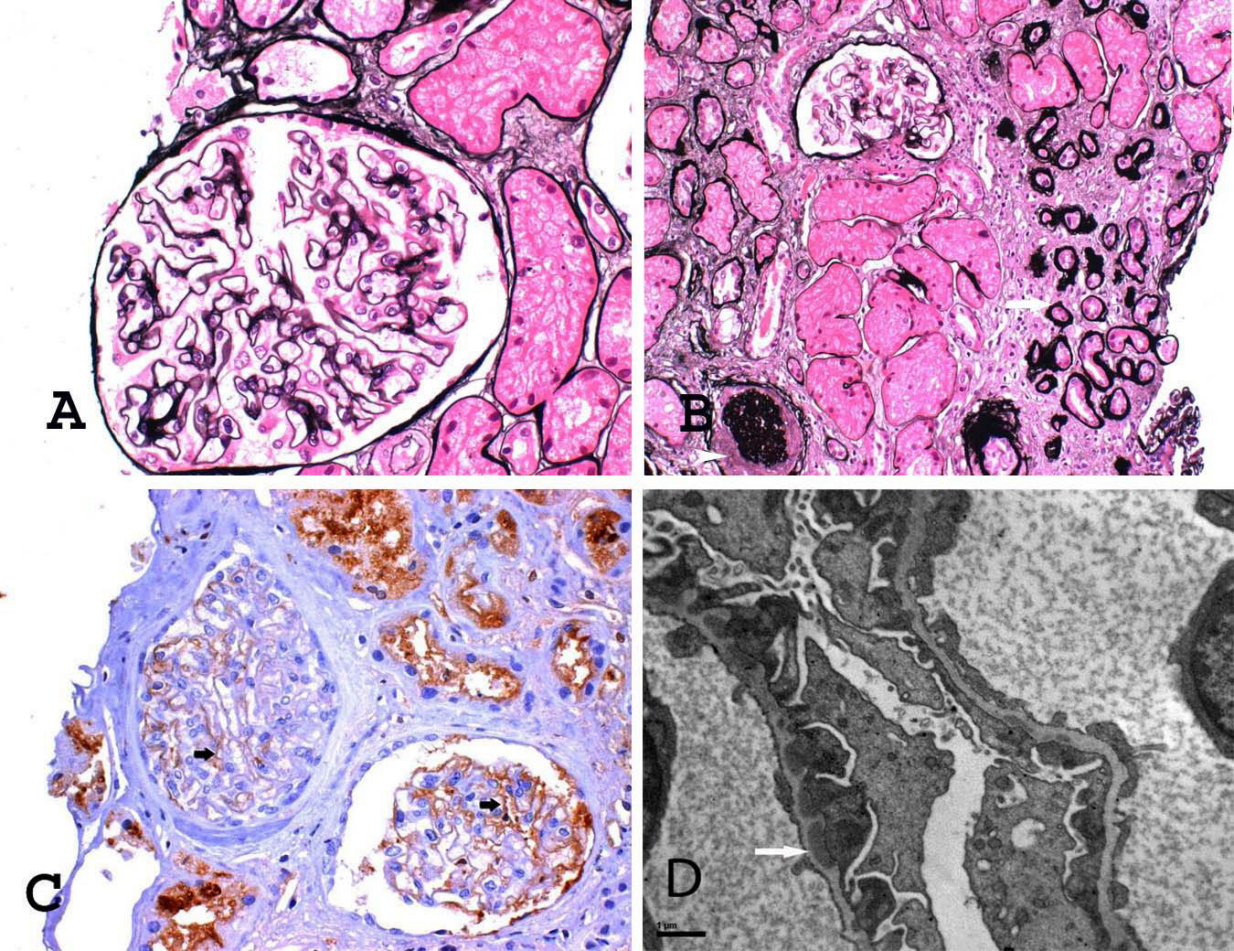
**A. Mild thickening and rigidity of the glomerular capillary walls with slight dilatation of the capillary lumens [JMSx400]; B. Focus of tubular atrophy and interstitial fibrosis (white arrow), global wrinkling and sclerosis of a glomerulus (white arrowhead) [JMSx200]; **C**. IgG immunohistochemical expression in glomerular capillary walls (black arrows) [IgGx400]; **D**. Sub-epithelial dense deposits (white arrow), effacement of epithelial foot processes and segmental spike formation (×13000)**.

A bone-marrow biopsy did not display any recurrence of the leukemia, while a chimerism analysis showed complete donor-type cells. At this point, a renal biopsy was performed to elucidate the underlying cause of proteinuria. The kidney specimen contained 31 glomeruli. Six of them were globally sclerotic and three had segmental sclerosis. The remaining 22 showed mild thickening of the capillary walls with segmental spike formation. Immunohistochemistry revealed diffuse deposits of IgG and C3 on the outer aspect of his glomerular capillary wall and segmental mesangial IgM deposits. Electron microscopy showed diffuse sub-epithelial deposits and foot processes fusion. These findings were consistent with MN stage I, accompanied by focal sclerotic lesions (Figure 1).

Complete resolution of the NS, improvement of his renal function (serum creatinine 168 μmol/L) and a restoration of pancytopenia were achieved within two months of treatment with cyclosporin (150 mg/d) and methylprednisolone (36 mg/day) with subsequent tapering.

Six months later, he manifested a relapse of acute leukemia. He refused to undergo a second transplant with a reduced intensity preparative regimen, so an attempt was made to achieve remission by chemotherapy alone. Unfortunately he died of sepsis one month later.

## Discussion

Most cases of NS post HCT develop a few months after minimization or cessation of a GVHD prophylaxis regimen [6-10]. The principal histological entity reported is MN followed by minimal change disease, while other diagnoses are very rare. Almost all patients have presented some kind of GVHD (acute or chronic) prior to the development of proteinuria. The treatment of the NS post HCT is achieved mainly by steroids and cyclosporin [6-10].

To the best of our knowledge, this is the first reported case of a patient with MN post HCT fulfilling the clinical and laboratory criteria for the diagnosis of SLE (pancytopenia, glomerulonephritis, high anti-dsDNA titres and hair loss). Another reported patient with positive anti-dsDNA antibodies was a 12-year old girl who developed minimal change disease (MCD) after an umbilical-cord blood transplantation, without other manifestations of SLE [11]. The presence of segmental IgM deposits in the mesangium supports the diagnosis of secondary MN which, in connection with the positive anti-dsDNA antibodies, raises the possibility of a membranous nephropathy secondary to lupus. Nevertheless, alopecia and immune cytopenias are both well-known features of cGVHD, while IgM deposits in the mesangium have been reported in few other cases of glomerulonephritis post HCT [7]. Moreover, the temporal association of proteinuria with recurrent cGVHD manifestations, the rapid resolution of NS after a short course of cyclosporin and steroids, the diffuse ANA staining pattern and the negative CLIFT test, reduce the possibility of a true lupus MN and make more likely the diagnosis of an MN secondary to cGVHD with associated lupus-like manifestations.

Finally, our case report also raises the possibility that a more intensive blockade of the renin-angiotensin system with less immunosuppression may be more appropriate for the treatment of NS post HCT.

## Conclusions

NS post HCT is a late complication usually presenting after withdrawal of GVHD prophylaxis therapy. cGVHD is characterized by the appearance of autoimmune phenomena similar, but not identical, to those seen in autoimmune diseases. The decision for more immunosuppression has to be weighed against the need for preservation of the graft versus leukemia phenomenon.

## Abbreviations

aGVHD: acute graft-versus-host disease; AML: acute myelogenous leukemia; ANA: anti-nuclear antibodies; ANCA: anti-nuclear cytoplasmic antibodies; cGVHD: chronic graft-versus-host disease; CLIFT: crithidia luciliae immunofluorescence test; CMV: cytomegalovirus; ELISA: enzyme-linked immunosorbent assay; HBV: hepatitis B virus; HCV: hepatitis C virus; HCT: hematopoietic cell transplantation; HIV: human immunodeficiency virus; HLA: human leukocytes antigen; HPF: high power field; MCD: minimal change disease; MN: membranous nephropathy; NS: nephrotic syndrome; SLE: systemic lupus erythematosus.

## Competing interests

The authors declare that they have no competing interests.

## Authors' contributions

KS, SS, VM, IP, DX, EV, SS, KP, TK, PK, IX, CP, MA, ED were involved in patient care in the hematology unit and the renal unit, acquisition of data, analysis and interpretation of data, review of the literature, and drafting and revising the manuscript. All authors read and approved the final manuscript.

## Consent

Written informed consent was obtained from the patient's next-of-kin for publication of this case report and any accompanying images. A copy of the written consent is available for review by the Editor-in-Chief of this journal.
